# Enhancing Titanium Osteoconductivity by Alkali-Hot Water Treatment

**DOI:** 10.1021/acsomega.4c06702

**Published:** 2024-10-22

**Authors:** Li Chang, Peng Chen, Takayuki Mokudai, Masakazu Kawashita, Itaru Mizoguchi, Hiroyasu Kanetaka

**Affiliations:** 1 Graduate School of Dentistry, Tohoku University, Sendai 980-8575, Japan; 2 Joining and Welding Research Institute, Osaka University, Osaka 567-0047, Japan; 3 Institute for Materials Research, Tohoku University, Sendai 980-8577, Japan; 4 Laboratory for Biomaterials and Bioengineering, Institute of Science Tokyo, Tokyo 101-0062, Japan; 5 Graduate School of Biomedical Engineering, Tohoku University, Sendai 980-8575, Japan

## Abstract

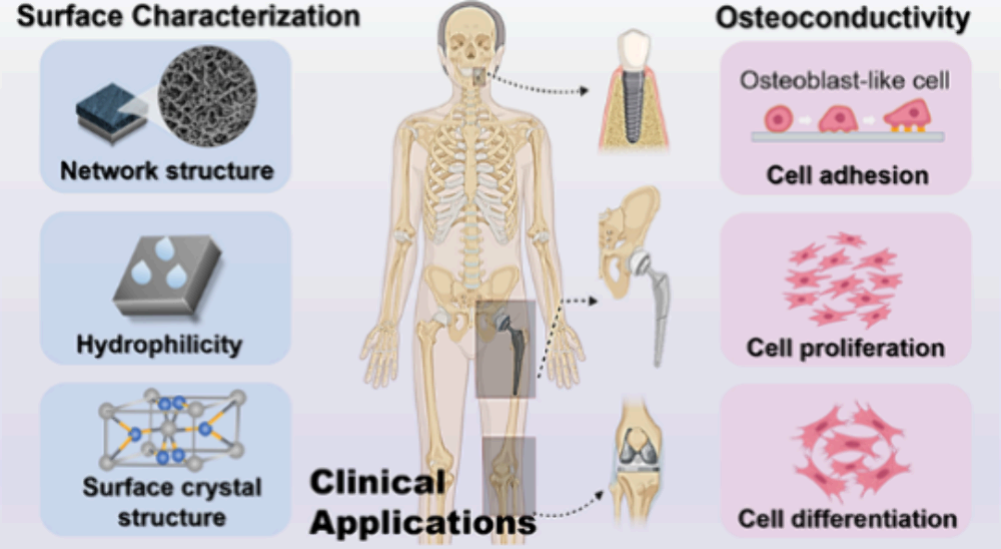

Titanium and its alloys are essential in orthopedic and dental treatments owing to their high strength, corrosion resistance, and superior biocompatibility compared with those of other metals. However, titanium alloys are bioinert. Previous studies have indicated that alkali treatment (AT) is a straightforward method to create a surface oxidization layer on titanium, thereby improving its bioactivity. Herein, alkali-hot water pretreatment was used to enhance the osteoconductivity of titanium and to identify a simple and efficient means of enhancing the interaction between osteoblasts and implants for clinical applications. Commercial pure titanium plates were ground (CP Ti) and subjected to alkali solution and hot water treatments (AWT). Single-process CP Ti specimens were prepared via either AT or hot water treatment (WT). Network-like structural features were observed in the AT specimens and were further refined and densified in the AWT specimens. Water contact angle testing revealed that the hydrophilicity of the titanium specimen (80° for CP Ti) increased by 19° for the AT specimens but decreased by 59° for the AWT specimens. Mouse preosteoblasts (MC3T3-E1 cells) were used for *in vitro* evaluation. After 24 h of culturing, the number of attached MC3T3-E1 cells on the AWT specimens was 1.5 times larger than that on the CP Ti specimens, suggesting that the alkali-hot water treatment enhanced the initial cell attachment. Cell proliferation evaluation indicated that fewer cells were detected in the AT and AWT specimens compared with those in the CP Ti or WT specimens. However, osteogenic differentiation evaluation on day 10 revealed a 1.5-fold higher alkaline phosphatase expression in cells cultured on the AWT specimens than in cells cultured on the CP Ti specimens. These findings demonstrate the good cytocompatibility and osteoconductivity of AWT Ti, highlighting its benefits in orthopedics and dental treatments.

## Introduction

With the increasing elderly population and high prevalence of osteoarthritis, osteoporosis, bone injury, and obesity, as well as the rising number of trauma cases from traffic and sports accidents, there has been a significant rise in knee and hip arthroplasty procedures. This burgeoning demand has prompted the orthopedic implant industry to set higher standards for the development of biomaterials, particularly those addressing improvements in corrosion resistance, biocompatibility, and reduction of material wear. Among the numerous available materials, titanium and its alloys, which are used for most implants, stand out because of their lightweight, low density, low elastic modulus, and high strength. The natural affinity of titanium to oxygen leads to the rapid formation of a thin, stable oxide layer on its surface when exposed to air or oxygen-rich environments, significantly enhancing its resistance to chemical attacks and corrosion.^1^ These properties provide implants a high damage tolerance, reduced bone resorption, and enhanced osseointegration.^2^ Therefore, titanium and its alloys are used widely in dental implants and orthodontic temporary anchorage devices (TADs) in dentistry. In contrast to orthopedic implants, dental implants are typically smaller. They are immediately subjected to strong masticatory forces, and are frequently exposed to the microbiologically active and mildly acidic environment of saliva,^3−6^ which leads to persistent demands for the better short-term stability and long-term performance of titanium implants. The successful anchorage of dental implants and TADs is contingent upon the quantity of bone that osseointegrates directly onto the titanium surface.^7^ However, the reported bone-to-implant contact (BIC) rate for dental implants is 53.0%–77.4%.^8^ The low BIC rate may be a reason for the failure to achieve osseointegration and early failure of dental implants. In addition, the failure to establish the formation of stable, early bone-implants can lead to aseptic loosening, which is a common cause of failure of cementless joint replacement surgery.^9^ These difficulties indicate that the osteoconductivity of current titanium implants still presents significant challenges.

To address these challenges, surface engineering has been applied to enhance the properties of titanium via surface modification, nanoengineering, and hybrid materials.^10−12^ These advancements in morphology, roughness, chemical composition, and crystalline structure considerably influence osteoblast adhesion, proliferation, and differentiation, thereby dictating the rate and degree of bone integration, and ultimately increasing implant success rates. For titanium-based implants, proper surface modification confers the specific characteristics required for osseointegration and preserves the intrinsic merits of the substrate material. Common titanium surface modification techniques include acid–alkali etching, sandblasting, and anodization. Alkaline etching is a common surface modification method for orthopedic and dental titanium implants.^13,14^ Alkali-treated titanium surfaces form a sodium hydrogen titanate layer that can precipitate a calcium phosphate layer in simulated body fluids (SBFs), thereby enhancing the bioactivity of the material.^15,16^

Previous studies have demonstrated that adjusting the alkali concentration and parameters of the subsequent heat treatment can alter the crystalline phase^17^ and hydrophilicity^18^ of materials, thereby enhancing their osteoconductivity. Furthermore, considering its straightforward, economical, and manageable procedure, alkaline treatment is a preliminary step in material modification studies. For instance, alkali treatment to facilitate drug loading and element doping on a network-like structure can achieve bone bonding^19^ and antibacterial effects.^20^ Expanding on this foundation, alkali-hot water treatment, which involves sequential applications of alkali and hot water, has been proposed to promote more effective apatite nucleation in SBFs, resulting in an enhanced bone-binding capacity.^21^ Nevertheless, the effects of the physicochemical property changes on osteoblastic behavior have not been fully elucidated.

Therefore, we hypothesized that the alkali-hot water treatment of titanium would enhance its bioactivity relevant to osseointegration. In this study, alkali, hot water, and alkali-hot water treatments were applied to ground titanium surfaces. Surface analyses of pure titanium, both before and after alkali and/or hot water treatment, were conducted to assess the changes in its morphology, roughness, hydrophilicity, and chemical and crystalline structures. To assess the *in vitro* effects, mouse osteoblast-like cell line (MC3T3-E1) cells were cultured on these surfaces to predict bone formation, implant stability, healing, and integration through initial osteoblast adhesion, proliferation, and differentiation. This study was conducted to elucidate the effects of the physicochemical characteristics of alkali-hot water-treated (AWT) titanium surfaces on their osteoconductivity, thereby providing insights for future clinical applications.

## Materials and Methods

### Sample Preparation

Commercially pure titanium specimens were purchased (JIS grade 2, 10.5 mm^2^, 1 mm thickness; Nihon Plate Seiko Co., Ltd.). The titanium specimens were ground in random directions using a #400 diamond pad, and subsequently washed via ultrasonication. Then, they were subjected to one of three treatment processes: immersion in a 5 M NaOH solution at 60 °C for 24 h (AT), immersion in hot water at 80 °C for 48 h (WT), or AWT. After being rinsed with ultrapure water, all the specimens were dried under vacuum and sterilized before use.

### Surface Morphology

The morphologies of the titanium surfaces were observed using scanning electron microscopy (SEM; JSM-6390LA; JEOL Ltd., Tokyo, Japan). Images were recorded using an accelerating voltage of 10 kV. The electron beam spot size was set to 40.

### Surface Roughness

The surface roughness of the titanium specimens was measured using a noncontact three-dimensional (3D) profiler (K505-232-02; Taylor Hobson Ltd., Leicester, UK) equipped with image analysis software (TalyMap Gold 6.2.6746). The mean surface roughness (Ra) was calculated for five randomly chosen areas (160 × 160 m) per specimen.^22^

### Surface Hydrophilicity

The hydrophilicity of the titanium surfaces was evaluated by measuring the water contact angle and surface energy. The contact angle was measured using a sessile drop instrument (PG-X-Plus; Matsubo Corp., Tokyo, Japan) equipped with a digital camera and image analysis software. Ultrapure water was used as the wetting liquid with a drop size of 5 μL. The contact angles of the air–water–substrate interface were measured five times for three specimens per group. The surface energy was calculated using the Young–Dupré equation according to the contact angle results.^23^

### Surface Chemical Functional Group

The functional groups of the prepared specimens were analyzed using Fourier-transform infrared spectroscopy (FT-IR; JASCO, FT/IR-4X, Tokyo, Japan) with a resolution of 4 cm^–1^ in the region of 4000–400 cm^–1^ and scan speed of 2 mm/s. After which, the data were calibrated through analysis software.

### Surface Crystal Structure and Phase Composition

Thin-film X-ray diffraction (TF-XRD; RINT-2200VL; Rigaku Corp., Tokyo, Japan) was used to analyze the crystal structures and phase compositions of the prepared specimens. These measurements were obtained with a voltage of 40 kV, current of 40 mA, scanning rate of 2°/min, sampling width of 0.02°, and measuring range of 3° ≤ 2θ ≤ 60°.

### Cell Culture and Induction of Differentiation

The MC3T3-E1 cell line, derived from mouse calvaria osteoblast precursors, was cultured on a polystyrene plate using Dulbecco’s modified Eagle’s medium (DMEM; Wako Pure Chemical Industries Ltd., Osaka, Japan) supplemented with 10% fetal bovine serum (FBS; Invitrogen Corp., Carlsbad, CA, USA) and penicillin–streptomycin (Meiji-Seika Kaisha Ltd., Tokyo, Japan) at 37 °C in a 5% CO_2_ humidified environment. For *in vitro* experiments, cells from the fourth passage were used after subculturing. Once the cells reached 80% confluence, they were detached using an ethylenediaminetetraacetic acid solution containing 0.5% trypsin (Gibco, NY, USA). Subsequently, the cells were seeded onto five sterilized titanium specimens at a density of 5000 cells/cm^2^, with tissue culture polystyrene (TCPS) as a reference for cell attachment. The cells were seeded in DMEM with the supplements for the initial cell adhesion experiment, and were cultured for 3, 6, or 24 h at 37 °C in 5% CO_2_. For the cell proliferation experiment, the cells were cultured under the same conditions for 1, 3, 5, or 7 d. The culture medium was changed every 2 d.

When the cells reached 100% confluence on the specimen surface, cell differentiation was induced by replacing the medium with DMEM containing 10% FBS and an osteoblast-inducer reagent (Takara Bio Inc., Otsu, Japan), including ascorbic acid, hydrocortisone, β-glycerophosphate, and bone morphogenetic protein 2 (PeproTech, Inc., NJ, USA). The cells were then cultured for 6, 8, 10, or 12 d under the same conditions. The culture medium was changed every 2 d.

### Immunofluorescence Staining

Immunofluorescence staining was used to observe cell adhesion. After incubation for 3, 6, or 24 h, the cells were fixed in 4% paraformaldehyde–formalin for 15 min, followed by three washes with phosphate-buffered saline (PBS; Fujifilm Wako Pure Chemical Corp.). The cells were permeabilized in 0.1% Triton X-100 for 10 min and then incubated in PBS containing 10 vol % goat serum albumin (#426042; Nichirei Corp., Tokyo, Japan) for 60 min to block nonspecific antibody reactions. The cells were then rinsed with PBS containing 10% Tween 20 (#11332465001; Roche Diagnostics GmbH, Germany). Then, they were incubated with a 1/100 dilution of vinculin monoclonal antibody (#33605–1-Ig; Sigma-Aldrich Corp., MO, USA) for 1 h at room temperature. After washing with PBS at least three times, the cells were incubated in a 1/400 diluted Alexa Fluor 488-conjugated goat antimouse IgG (Invitrogen Corp.) secondary antibody solution at room temperature for 1 h. Thereafter, the cells were incubated in a 7/1000 dilution of rhodamine phalloidin (Cytoskeleton Inc., Denver, CO, USA) for 30 min to stain F-actin. After being washed three times, the cells were mounted on a glass-bottom dish with a mounting agent containing 4′,6-diamidino-2-phenylindole for staining cell nuclei (H-1500; Vector Laboratories, Inc., Burlingame, CA, USA) and were observed using a model laser microscope. Cell morphometry was applied to the confocal laser microscopy images using ImageJ software (National Institutes of Health, Bethesda, MD, USA). For the initial cell adhesion counting, five images were randomly captured at a magnification of 10× for each sample. In total, 20 regions were analyzed across the three specimens in each group. To assess the initial cell adhesion morphology, 300 cells from each group were randomly captured at a magnification of 20×. The number, area, aspect ratio, and circularity of the attached cells were quantified using the ImageJ software.

### Cell Proliferation

After the desired proliferation and differentiation periods, all the specimens were transferred to 24-well polystyrene culture plates. The cells were then rinsed with PBS. After the undifferentiated cells were counted, they were seeded and evenly distributed across the wells of a 24-well TCPS plate at the desired experimental cell density. Subsequently, the cell counting Kit-8 reagent (Dojindo Laboratories, Kumamoto, Japan) was added to each well according to the manufacturer’s instructions. The plate was then incubated at 37 °C for 3 h. Following incubation, the absorbance of the converted formazan product was measured at 450 nm using a microplate spectrophotometer (GloMax-Multi Detection System; Promega Corp., Tokyo, Japan). A standard curve was generated based on the absorbance values of the respective cell numbers. The number of cells in each sample was estimated using a standard curve.

### Cell Differentiation

After 6–12 d of differentiation, the cells were rinsed with PBS. Then, after 300 μL of alkaline phosphatase yellow liquid substrate (P7998; Sigma-Aldrich Corp.) and 3 μL of 10% Triton-X/PBS were added to each well, the plate was incubated at 37 °C for 15 min. Once the yellow color was developed, the reaction was stopped by adding 2 M NaOH. The absorbance was measured at 405 nm using the microplate spectrometer. Based on the WST-8 standard curve, the alkaline phosphatase (ALP) expression per square centimeter of each sample was estimated. This method enables the standardization and comparison of ALP expression across different specimens and conditions, considering the variations in cell numbers.

### Statistical Analysis

All the results presented herein were obtained from at least three independent experiments, with each experiment having a sample size of at least 15. All the data are presented as the mean ± standard deviation. The data were evaluated using analysis of variance. Significant differences between the groups were determined using Bonferroni’s modification of the Student’s *t test*. The statistical significance was set at *P* < 0.05. Otherwise, the results were considered insignificant.

## Results and Discussion

### Topographic and Physicochemical Characteristics of Titanium Surfaces

**Figure 1:**
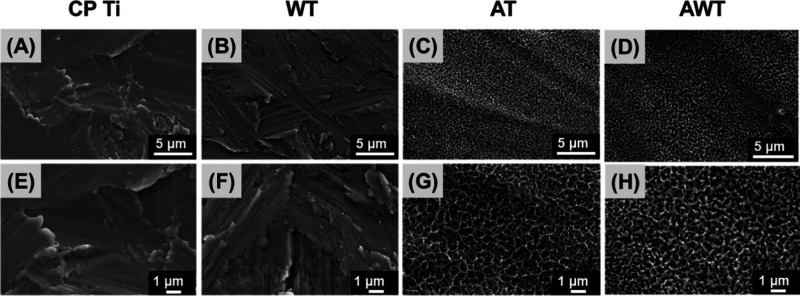
Surface morphologies of all the specimens shown in SEM images: (A, E) commercially obtained pure titanium (CP Ti); (B, F) alkali-treated (AT); (C, G) hot water-treated (WT); and (H, I) alkali-hot water-treated (AWT). Scale bars are 5 μm (A–D) and 1 μm (E–H).

The surface topography of each specimen was qualitatively evaluated based on the SEM images. These findings confirmed that alkali treatment imparts structural features to titanium surfaces. The CP Ti starting specimens exhibited randomly oriented grinding structures (Figure 1A,E), resembling the results obtained for the WT specimens (Figure 1B,F). At a low magnification (Figure 1C,D), the SEM analyses revealed a similar network-like microscale topography for the AT and AWT specimens. However, a higher magnification (Figure 1G,H) revealed that the AWT surfaces had a more compact reticular arrangement. Previous studies have confirmed that suitable microscale morphological changes can strongly affect cell behavior and enhance cell adhesion, migration, proliferation, and differentiation.^22,24,25^

In this study, the titanium surface was subjected to alkali etching, forming a surface network-like structure (Figure 1), which increased the surface area and complexity of the microenvironment and provided cells with more opportunities for physical attachment and communication. The subsequent hot water treatment refined the network-like structure. This change potentially influences the directional growth and migration of cells.^26^

**Figure 2:**
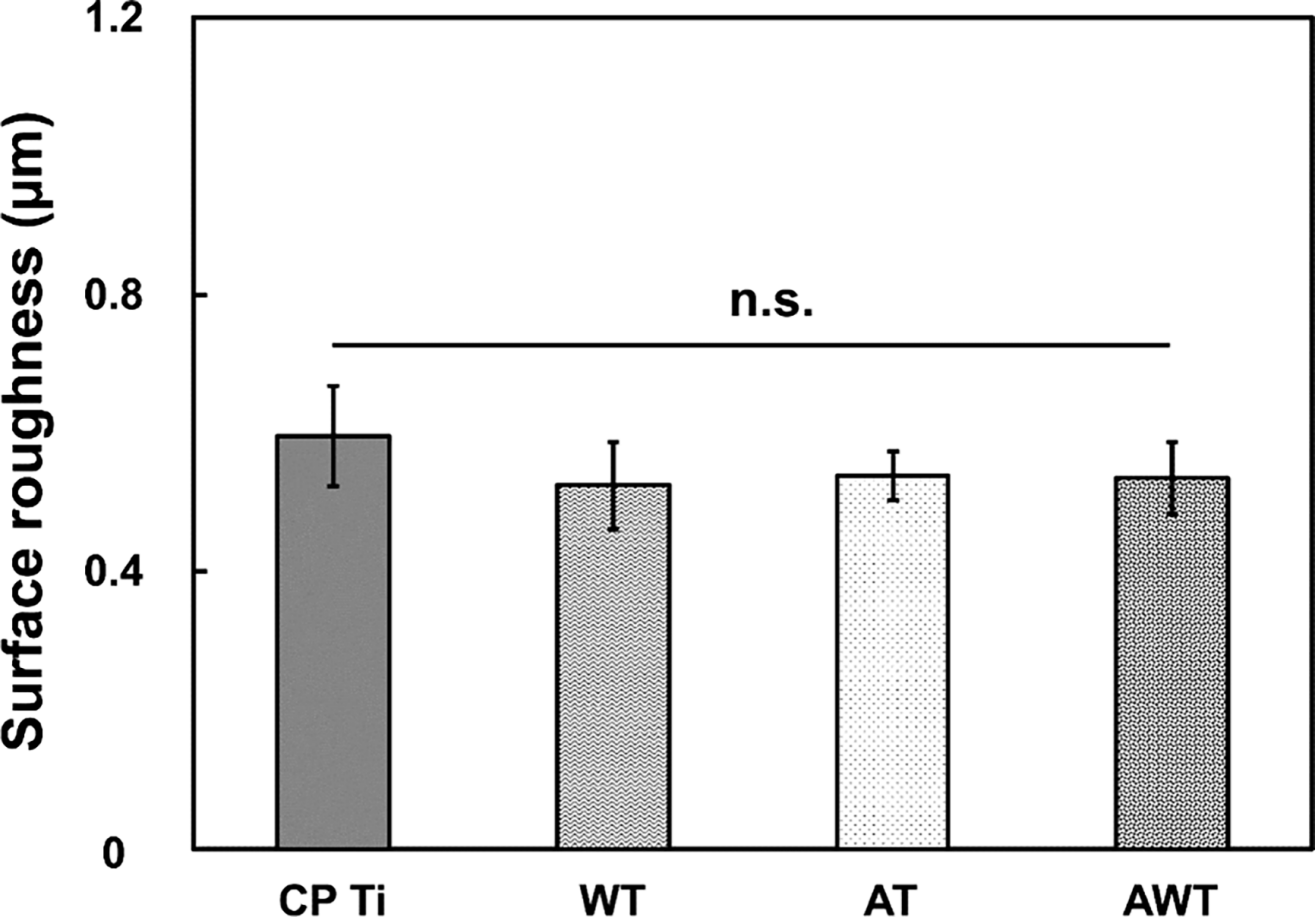
Surface roughness of all the specimens measured using a 3D noncontact optical surface profilometer. Vertical roughness parameters: arithmetical mean height (Sa). Data are presented as mean ± standard deviation. n.s., not significant.

The topographic characteristics of the specimens were quantitatively measured using a 3D profiler (Figure 2). Notably, despite the observed changes in the surface topography, the surface roughness measurements of the AT and AWT specimens were consistent with those of the CP Ti and WT specimens. This consistency highlights that the microscale morphology introduced by the alkali treatment did not drastically alter the surface roughness (Figure 2). Some studies have recommended increased roughness to enhance cell adhesion.^27,28^ However, notably, bone integration does not constantly improve with increased surface roughness.^29^ Therefore, intentionally increasing the roughness may not be the best strategy. Considering that surface roughness can affect the wetting behavior and subsequent interaction between the implant surface and its biological environment,^30^ this study excluded the influence of roughness on wettability by maintaining consistency in roughness among the treatments.

**Figure 3:**
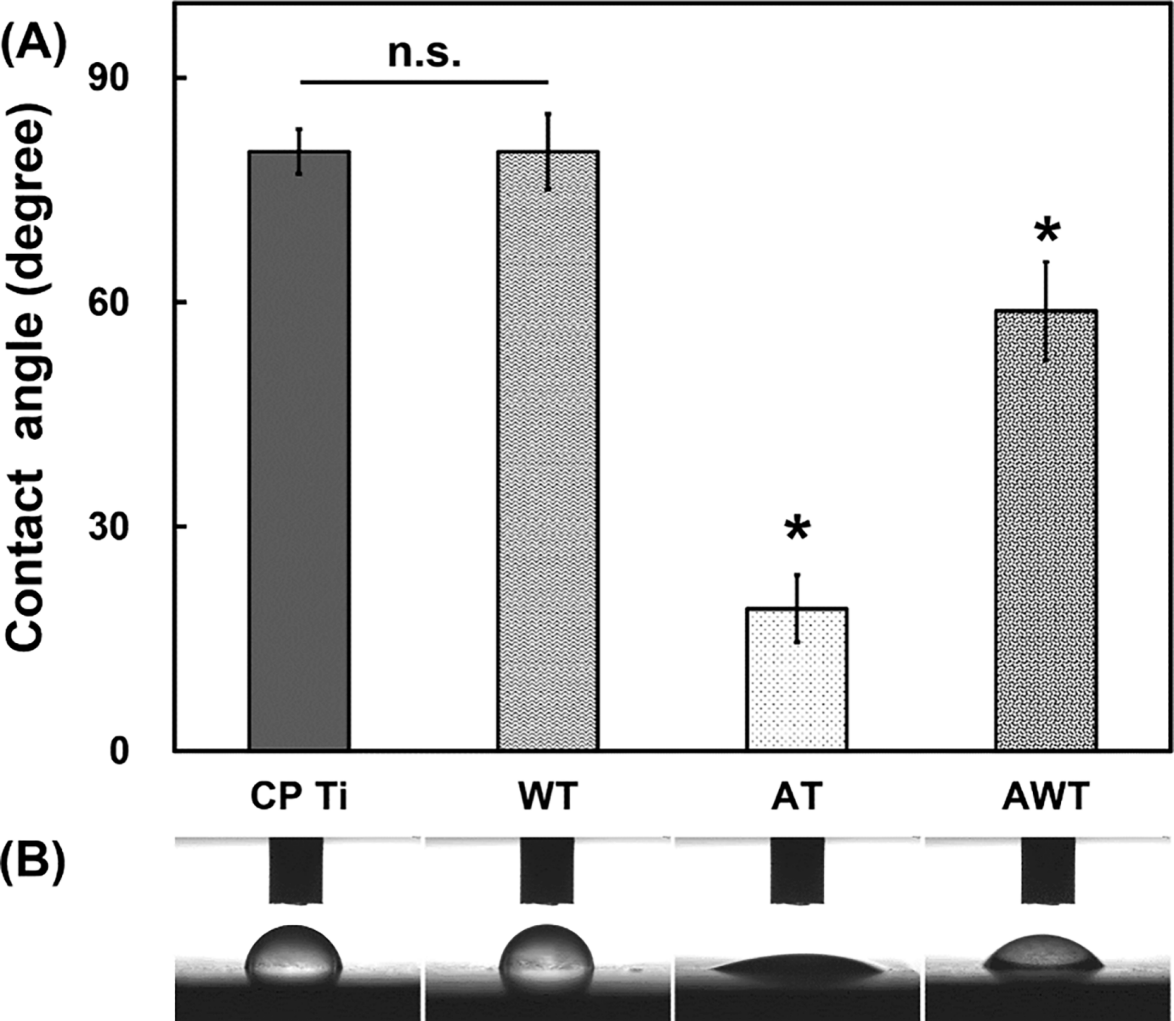
Wettability of the titanium specimens: (A) water contact angles and (B) corresponding photographs of water droplets on different titanium surfaces. Data are presented as mean ± standard deviation. n.s., not significant; *, *P* < 0.05.

Water contact angle measurements indicated that all the titanium specimens had contact angles <90°, reflecting relative hydrophilicity.^27,28^ Specifically, the AT (19.03° ± 4.47°) and AWT (58.83° ± 6.55°) specimens had considerably smaller contact angles than the CP Ti (80.14° ± 2.97°) and WT (80.14° ± 5.04°) specimens, which confirmed that the hydrophilicity was enhanced with alkali treatment (Figure 3).

By substituting the measured contact angles into the Young–Dupré equation, we calculated the surface energy of each sample (Table 1). The results showed that the surface energy of the titanium surface significantly increased after alkali treatment, particularly in the AT group. High-surface-energy materials have strong attraction to water; consequently, water molecules are more easily attracted to and spread across the surface, thereby reducing the contact angle.

**Table 1:** Surface Roughness, Water Contact Angle, and Surface Energy Data for Titanium Specimen Surfaces

**condition**	**Sa (μm)**	**water contact angle (deg)**	**surface energy (mN/m)**
CP Ti	0.55 ± 0.06	80.14 ± 2.97	85.25 ± 3.72
WT	0.54 ± 0.08	80.14 ± 5.04	85.21 ± 6.24
AT	0.54 ± 0.05	19.03 ± 4.47^a^	141.41 ± 1.94^a^
AWT	0.54 ± 0.07	58.83 ± 6.55^a^	110.26 ± 7.36^a^

a (*P* < 0.05): results were indicated as statistically significant compared with other conditions.

**Figure 4:**
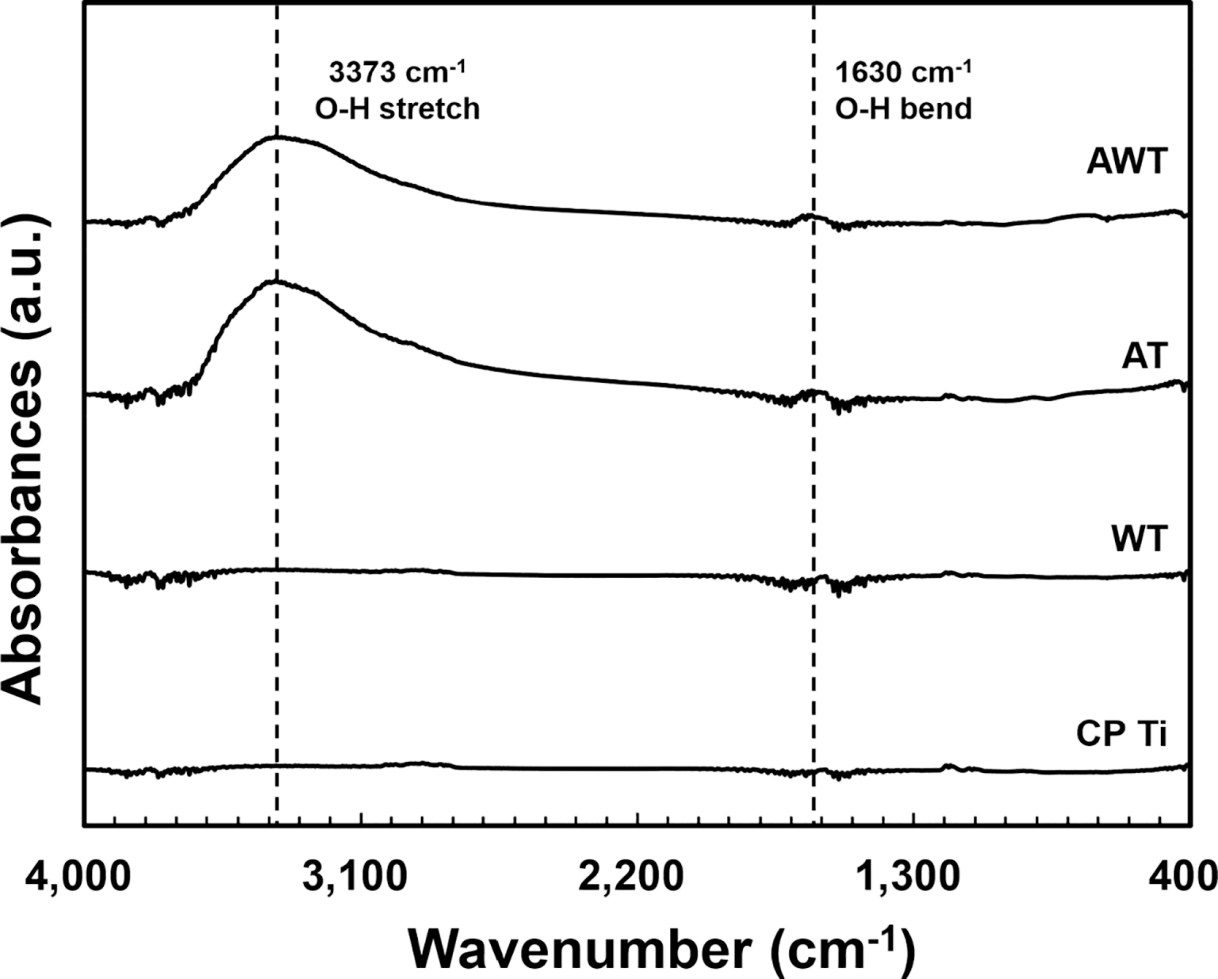
FT-IR spectra of the titanium specimen surfaces.

The FT-IR spectra shown in Figure 4 reveal characteristic absorption bands for functional groups. After alkali treatment (AT and AWT groups), the absorption peak around 3373 cm^–1^ significantly increased, whereas the absorption peak around 1630 cm^–1^ showed a slight increase. The broad band around 3373 cm^–1^ is attributed to the fundamental stretching vibrations in hydroxyl groups (O–H), which may be due to the absorption of water from the atmosphere and formation of Ti–OH bonds. Additionally, the band at 1630 cm^–1^ is related to the bending vibrations of −OH.^31,32^ These results for the hydroxyl groups are consistent with the significant increase in hydrophilicity observed on the surfaces of the AT titanium specimens, indicating that the hydroxylation of the titanium surface contributed to the enhanced hydrophilicity. Notably, the alkaline hydroxyl groups on the oxide layer of the titanium surface may play an important role in the chemical interactions between the osteoblasts and titanium surface.^33^

**Figure 5:**
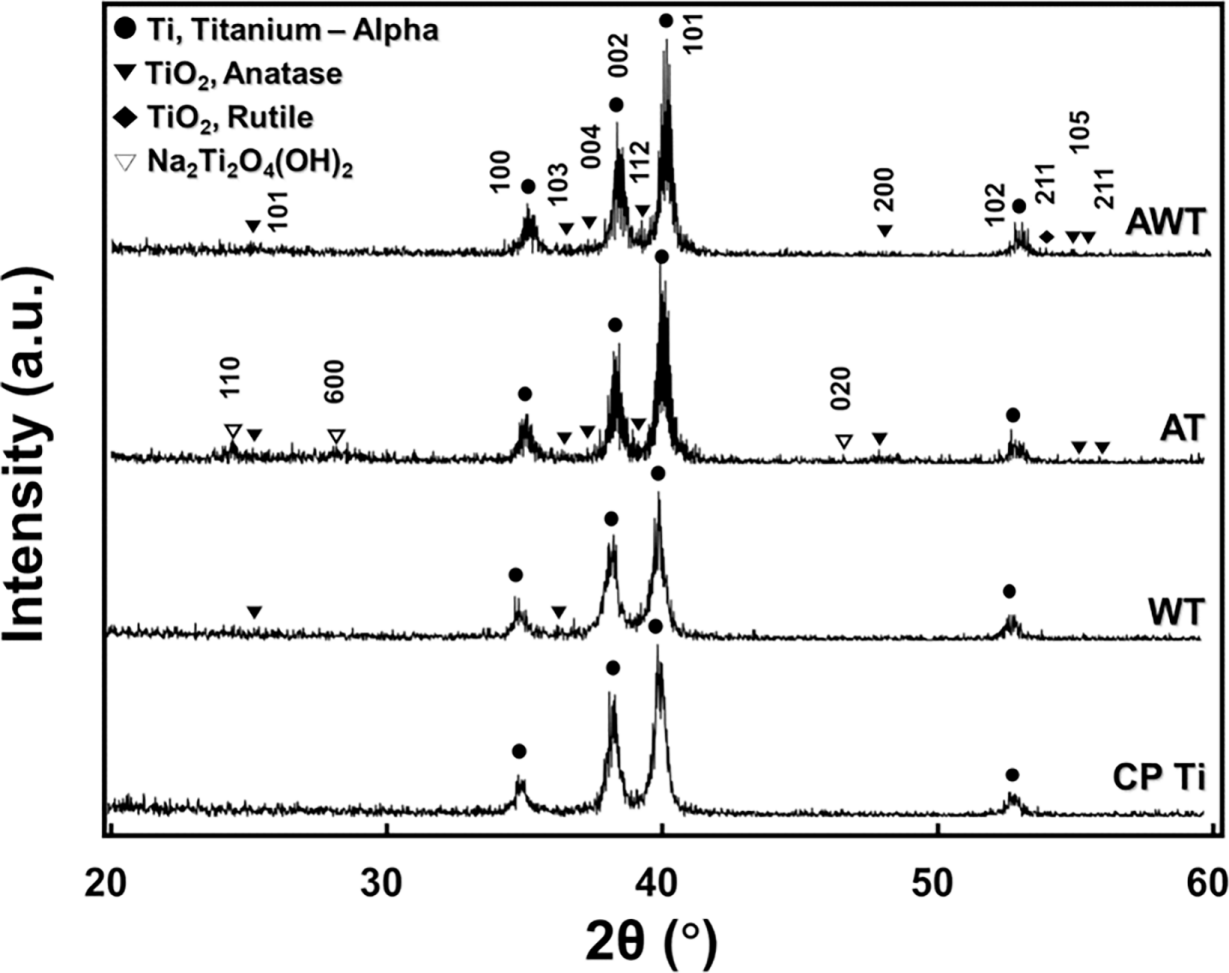
TF-XRD patterns of the titanium specimen surfaces.

Figure 5 shows the TF-XRD patterns of the analyzed specimens. All the titanium specimens exhibited analogous diffraction peaks associated with α-titanium (PDF #97-004-3416). Additionally, minor amounts of anatase TiO_2_ (PDF #00-021-1272) and rutile TiO_2_ (PDF #00-021-1276) were discernible in all the treated titanium specimens. Distinctive peaks corresponding to sodium titanium oxide hydroxide (Na_2_Ti_2_O_4_(OH)_2,_ PDF #00-057-0123) were observed in the AT group but not in the AWT group, indicating that the titanate formed via the alkali treatment was removed during the hot water treatment. Amorphous sodium titanate can interact with H_3_O^+^ ions in water, producing Ti–OH groups, which are integral to protein adsorption during cell adhesion,^30^ while improving the surface hydrophilicity of the material. This finding was consistent with the decrease in hydrophilicity after titanate removal in the AWT group. However, both *in vivo* and *in vitro* studies have demonstrated that the removal of sodium titanate increases the surface biological activity. An animal study reported that Na^+^ removal can amplify the bioactivity of bioactive titanium, consequently aiding bone-implant stabilization *in vivo*.^34^ Although an *in vitro* study has emphasized that anatase formed after the hot water treatment of sodium titanate gel enhances apatite formation capabilities in SBFs,^21^ very few titanium dioxide crystals, such as anatase and rutile, were observed during the experiments conducted for this study. This variation may be attributed to the differences in the brand and purity of the commercially pure titanium specimens used in this study.

Thus, the effects of the changes in the physicochemical properties on osteoconductivity during AWT are complex and interesting. Therefore, to provide theoretical support for the clinical application of this treatment, further exploration of the specific effects of each step of the treatment process on osteoblasts is required.

### Effects of Titanium Surfaces on the Early Adhesion and Morphology of Preosteoblasts

Cell adhesion is an intricate process that strongly affects the biocompatibility of biomaterials and supports cell survival, proliferation, and differentiation by coordinating various intracellular signals in response to external stimuli. The basis of these dynamic interactions lies in the dynamic interface formed between a cell’s membrane proteins and the adjacent extracellular matrix or underlying substrate.^35,36^

**Figure 6:**
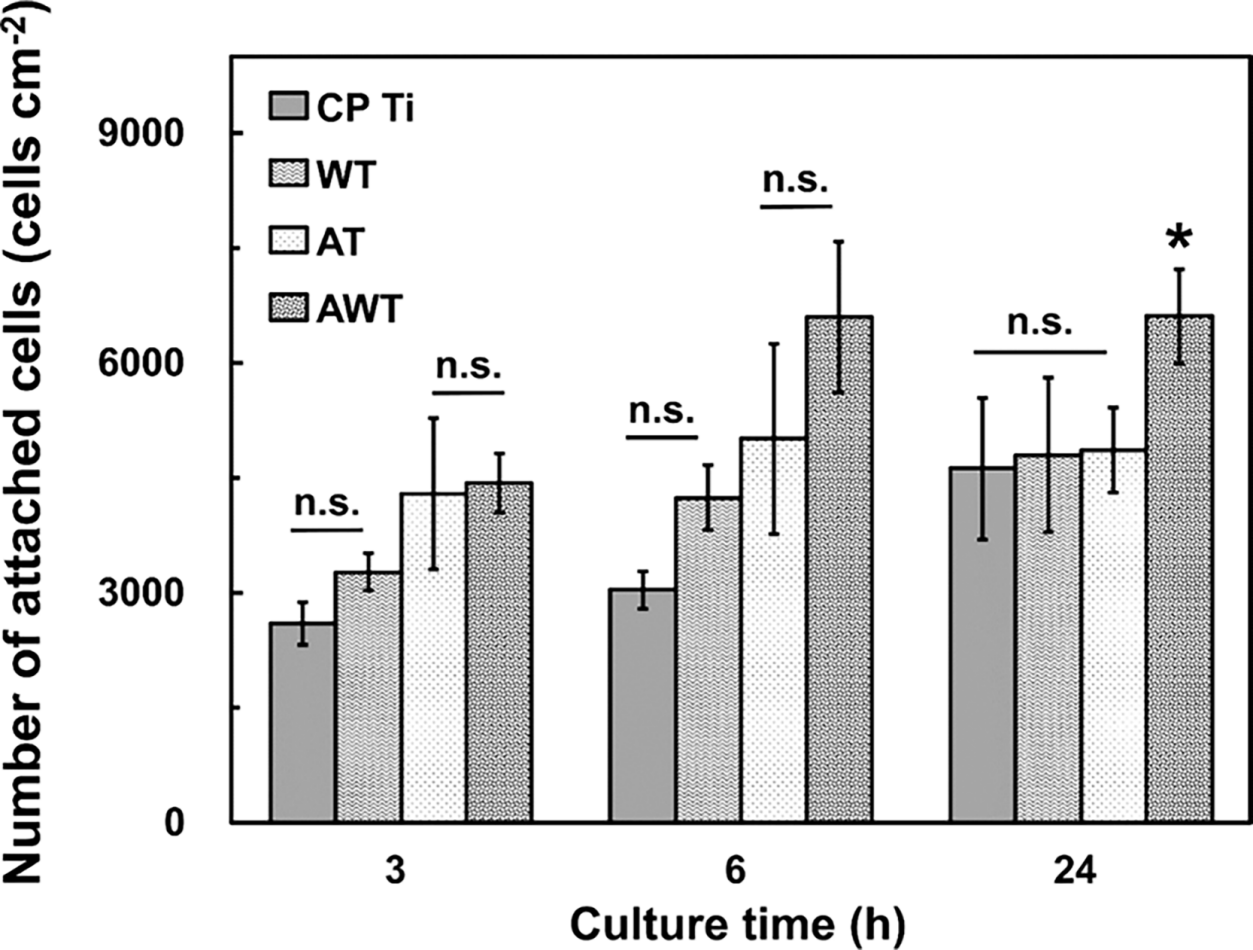
MC3T3-E1 cells attached to the titanium specimens after 3, 6, and 24 h of cell seeding. Data are presented as mean ± standard deviation. n.s., not significant; *, *P* < 0.05.

Figure 6 shows the number of MC3T3-E1 cells attached to the CP Ti, WT, AT, and AWT cells after 3, 6, and 24 h. During these periods, the CP Ti and WT groups exhibited fewer attached cells, whereas the AT and AWT groups exhibited larger counts. Additionally, the cell adhesion peaks for the AT and AWT groups occurred sooner (between 6 and 24 h). At 24 h, the number of attached cells for AWT was 1.5 times greater than that observed for the other groups, indicating that the AWT improved cell attachment. These findings indicate that osteoblasts adhered to the surfaces of the AT titanium specimens more quickly, which can be attributed to their network-like morphology and improved hydrophilicity (Figure 6). The network structure provides the cells with more active sites for attachment. Moreover, strongly alkaline conditions remove hydrocarbons from titanium surfaces, thereby forming alkaline hydroxyl groups, boosting the polar components on the titanium oxide film, enhancing protein adsorption rates, and consequently promoting cell sedimentation.^37^

**Figure 7:**
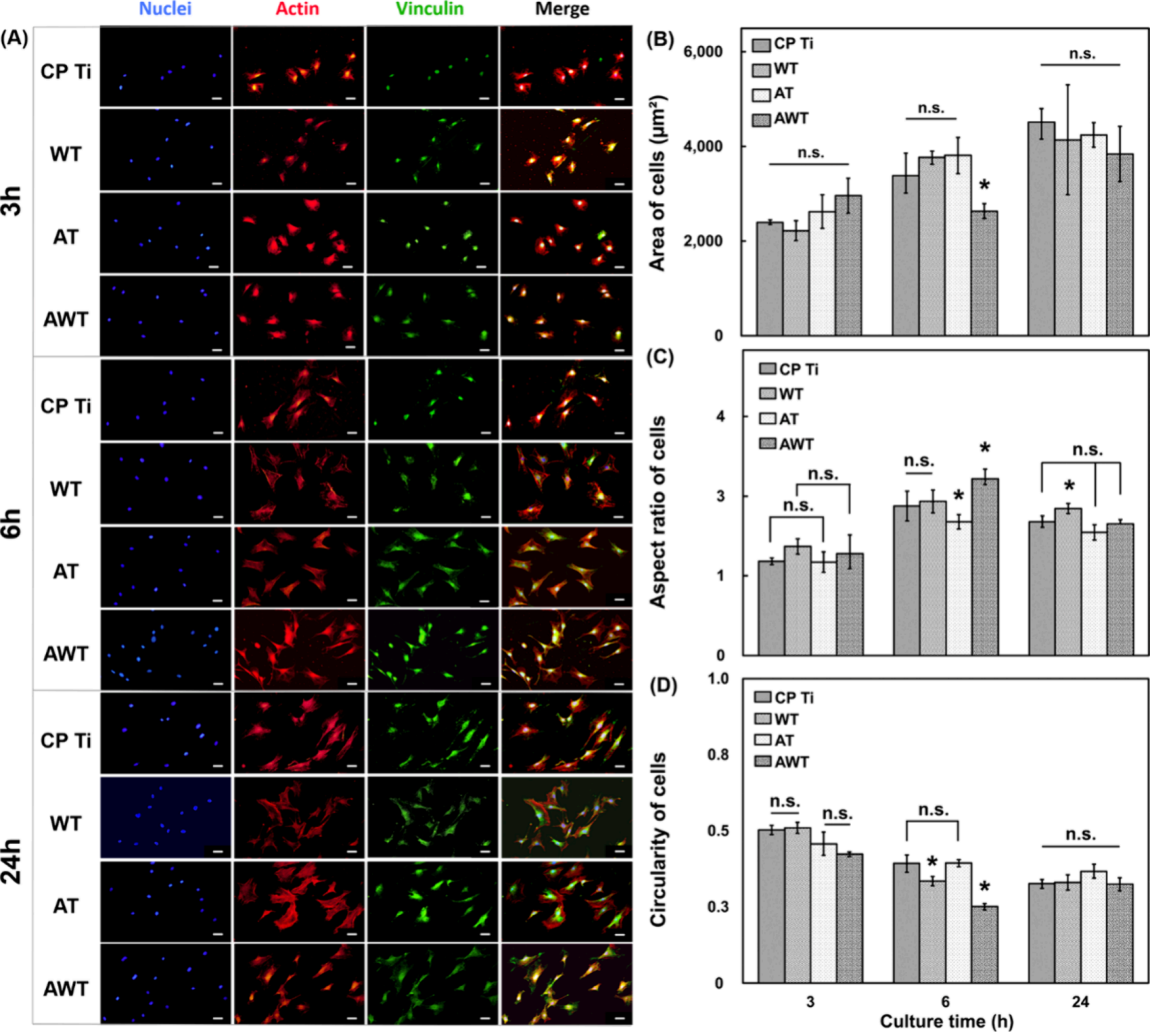
Morphology of MC3T3-E1 cells attached to the titanium specimens after 3, 6, and 24 h of cell seeding. (A) Electron microscope images of nuclei, actin, and vinculin staining of the MC3T3-E1 cells. Scale bars are 50 μm. (B–D) Cytomorphometric parameters, including the area, aspect ratio, and circularity, quantified for each image using the ImageJ software. Data are presented as mean ± standard deviation. n.s., not significant; *, *P* < 0.05.

Figure 7 shows the cellular morphologies (Figure 7a) and parameters (Figure 7b–d) of the MC3T3-E1 cells adhering to the titanium specimens. At 3 h, during the early phase of cell attachment, all the groups exhibited smaller cell areas. Concurrently, membrane-cytoskeletal vinculin predominantly clustered near the cell nucleus, marking the early stages of focal adhesion to the titanium substrate. However, the WT and AWT groups displayed cells with a slightly extended major axis, indicating that the preliminary connections between the cells and materials were advanced. The cells quickly recognize and adapt to the surface, which might facilitate better cell spreading.^38^ The cells adhered to the surfaces of the AT specimens might have been subjected to a strong adsorption force on the hydrophilic surface at this time, which restricted their directional movement. At 6 h, the cell polarity was enhanced. The cells on all the titanium surfaces exhibited more extensive and pronounced elongation. F-actin stress fibers appeared to be increasingly distinct and organized. Notably, only the cells in the AWT group showed no increase in surface area, but exhibited marked elongation in their major axis and decreased roundness. This behavior indicated that, by 6 h, the cells in the AWT group demonstrated superior multidirectional stretching compared with the other groups, closely followed by the WT and AT groups. Multidirectional stretching is crucial for cell movement and communication. Although reduced hydrophilicity may have hindered the adsorption capacity of the cells on the surfaces of the AWT specimens, it enhanced their migratory and communication capabilities, encouraging intercellular connections. At 24 h, the cells on all the surfaces exhibited increased spreading and clustering. However, this effect was most pronounced in the cells adhering to the surfaces of the AWT specimens, where the distribution of focal vinculin adhesions appeared more uniform and consistent. Vinculin plays an important role in cell adhesion by transmitting extracellular guidance signals to intracellular receptors.^39^ This finding suggests that the cells on the surfaces of the AWT specimens quickly develop stable adhesion and execute their potential biological functions.

### Effects of Titanium Surfaces on Cellular Proliferation

**Figure 8:**
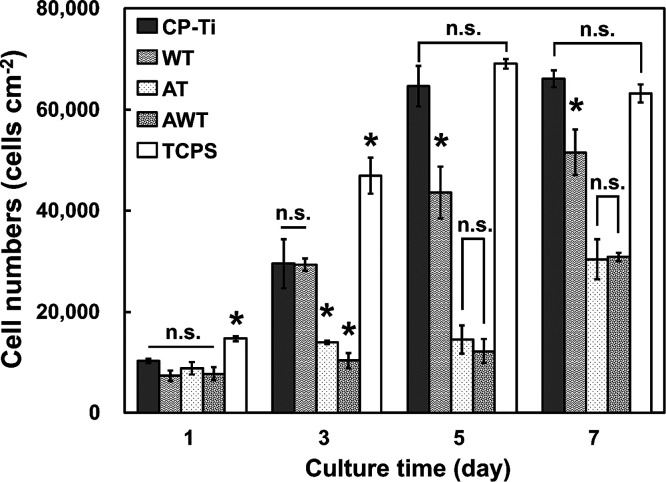
Proliferation of the MC3T3-E1 cells cultured on different specimens at days 1, 3, 5, and 7. Data are presented as mean ± standard deviation. n.s., not significant; *, *P* < 0.05.

Weak cellular proliferation was assessed quantitatively (Figure 8), and the TCPS group was chosen as the control. During a week, the number of MC3T3-E1 cells on all the surfaces increased, demonstrating the sustained biocompatibility of the titanium-based materials before and after surface modification. The lower cell proliferation in the AT group can also be linked to variations in surface chemistry and wettability. Furthermore, the hydrolysis of sodium titanate elevates pH levels at the cell–material interface, causing cellular alkalosis, which potentially inhibits cell proliferation and vitality.^40^ Moreover, fibronectin-mediated cell proliferation on hydrophobic surfaces occurs more rapidly than that on hydrophilic surfaces.^41^ The cell proliferation in the AT and AWT groups was slow but sustained (continuing until day 7), whereas that in the CP Ti and WT groups became stabilized by day 5. This suggests that, despite the slow initial growth rate, the cells on the surfaces of the AT and AWT groups realized a better proliferation potential on the day 7, which may indicate that these treatments provide a microenvironment more suitable for long-term cell attachment and growth.

### Effects of Titanium Surfaces on Osteogenic Differentiation

**Figure 9:**
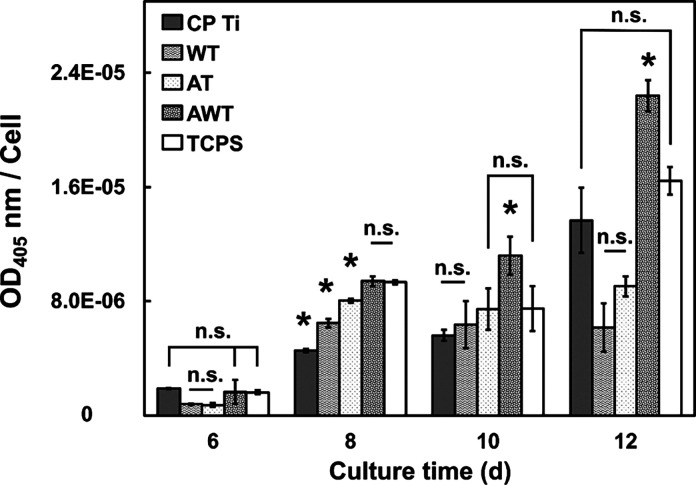
Osteogenic differentiation of the MC3T3-E1 cells cultured on different specimens. The ALP activity levels of each cell were evaluated through absorbency on days 6, 8, 10, and 12 after incubation. Data are presented as mean ± standard deviation. n.s., not significant; *, *P* < 0.05.

ALP expression, an indicator of osteogenic differentiation, was assessed on days 6, 8, 10, and 12 (Figure 9). In this study, the differentiation results were opposite to the proliferation results, which is consistent with the established principle that cell proliferation and differentiation are usually inversely related.^37^ Specifically, when osteoblast proliferation is inhibited, a concurrent increase in the expression of ALP occurs.

The ALP level in the AWT group was always higher than that in the AT and WT groups throughout the week. On days 6 and 8, the ALP expression in the AT and AWT groups was higher than that in the CP Ti and WT groups, indicating that the cells on the AT titanium surfaces had better osteogenic differentiation. However, the sharp increase in the ALP expression in the CP Ti, AWT, and TCPS (control) groups on day 12 was much higher than that on day 10, possibly owing to the formation of a continuous cell layer. As shown in Figure 8, the first peak time of ALP expression during the differentiation of single osteoblasts was inconsistent. For the CP Ti group, it was approximately on days 10 and 11. In the WT group, this was observed on days 8 and 9. This was observed approximately on day 8 in the AT group. The AWT and TCPS groups were assessed on day 8 or earlier. Consequently, the osteogenic expression intensity across the four titanium surfaces was sequenced as follows: AWT > AT > WT > CP Ti.

The excellent osteogenic differentiation promoted by the AWT surface is consistent with our previously described conclusions regarding the initial adhesion of osteoblasts, indicating that the AWT titanium surface characteristics are not conducive to the initial attachment of osteoblasts. These characteristics also positively influenced the subsequent differentiation processes. This influence may be attributable to the specific chemical and physical properties of the AWT surface, such as surface roughness, wettability, and chemical composition, all of which can affect cellular behavior. Additionally, Li et al. reported that AT titanium surfaces exhibit superior expression of osteogenic differentiation markers, including osteopontin and osteocalcin.^40^ This finding aligns with our conclusions.

### Implications for Orthopedic and Dental Implants

Challenges, such as aseptic loosening and implant failure owing to poor bone-implant formation, must be addressed when administering dental and orthopedic bone-implant treatments. Our study suggests that subjecting titanium to AWT augments its adhesion to osteoblasts and protein absorption without compromising cell vitality, thereby ensuring sustained osteoblast cell proliferation and effective differentiation. Data obtained from experiments have underscored the importance of AWT in strengthening bone–titanium bonding. Considering the efficiency, simplicity, and cost-effectiveness of this approach, its widespread adoption in dental and orthopedic treatments is anticipated. However, future studies must be conducted to evaluate the bone-bonding capability of this approach in a living body, as factors, such as long-term stability, biocompatibility, and the body’s biological responses, still need to be considered.

## Conclusions

Alkali-hot water pretreatment significantly enhanced the osteoconductive properties of titanium surfaces. In the *in vitro* study, the treated titanium exhibited increased wide osteoblast-like cell adhesion, elevated cell adhesion protein adsorption, and improved cellular motility and communication within 3–24 h. Despite a slower proliferation rate during the first week, the cells exhibited the potential for sustained proliferation. This treatment boosted the speed and efficacy of osteoblast differentiation, which are pivotal for orthopedic and dental implants. This study demonstrated the increased bioreactivity of AT and subsequently WT titanium. This bioreactivity is attributable to the suitable morphology, wettability, and surface physicochemical composition, but not to surface roughness. Overall, our findings highlight the benefits of alkali and hot water treatments in promoting the osseointegration of titanium implants, and suggest new strategies for orthopedic and dental applications. Future studies should examine the long-term performance *in vivo.*

## References

[ref1] Textor M.; Sittig C.; Frauchiger V.; Tosatti S.; Brunette D. M. Properties and Biological Significance of Natural Oxide Films on Titanium and Its Alloys. In Titanium in Medicine; Engineering Materials; Springer: Berlin Heidelberg: Berlin, Heidelberg, 2001; pp 171–230. 10.1007/978-3-642-56486-4_7.

[ref2] Jiang P.; Zhang Y.; Hu R.; Shi B.; Zhang L.; Huang Q.; Yang Y.; Tang P.; Lin C. Advanced Surface Engineering of Titanium Materials for Biomedical Applications: From Static Modification to Dynamic Responsive Regulation. Bioact. Mater. 2023, 27, 15–57. 10.1016/j.bioactmat.2023.03.006. 37035422 PMC10074421

[ref3] Ferrario V. F.; Sforza C.; Serrao G.; Dellavia C.; Tartaglia G. M. Single Tooth Bite Forces in Healthy Young Adults. J. Oral Rehabil. 2004, 31 (1), 18–22. 10.1046/j.0305-182X.2003.01179.x. 15125591

[ref4] Lafaurie G. I.; Sabogal M. A.; Castillo D. M.; Rincón M. V.; Gómez L. A.; Lesmes Y. A.; Chambrone L. Microbiome and Microbial Biofilm Profiles of Peri-Implantitis: A Systematic Review. J. Periodontol. 2017, 88 (10), 1066–1089. 10.1902/jop.2017.170123. 28625077

[ref5] Barão V. A. R.; Mathew M. T.; Assunção W. G.; Yuan J. C.; Wimmer M. A.; Sukotjo C. Stability of cp- Ti and Ti −6 Al −4 V Alloy for Dental Implants as a Function of Saliva pH – an Electrochemical Study. Clin. Oral Implants Res. 2012, 23 (9), 1055–1062. 10.1111/j.1600-0501.2011.02265.x. 22092540

[ref6] Kunrath M. F.; Dahlin C. The Impact of Early Saliva Interaction on Dental Implants and Biomaterials for Oral Regeneration: An Overview. Int. J. Mol. Sci. 2022, 23 (4), 2024 10.3390/ijms23042024. 35216139 PMC8875286

[ref7] Albrektsson T.; Brånemark P.-I.; Hansson H.-A.; Lindström J. Osseointegrated Titanium Implants: *Requirements for Ensuring a Long-Lasting, Direct Bone-to-Implant Anchorage in Man*. Acta Orthop. Scand. 1981, 52 (2), 155–170. 10.3109/17453678108991776. 7246093

[ref8] Le Guehennec L.; Goyenvalle E.; Lopez-Heredia M.; Weiss P.; Amouriq Y.; Layrolle P. Histomorphometric Analysis of the Osseointegration of Four Different Implant Surfaces in the Femoral Epiphyses of Rabbits. Clin. Oral Implants Res. 2008, 19 (11), 1103–1110. 10.1111/j.1600-0501.2008.01547.x. 18983312

[ref9] Abu-Amer Y.; Darwech I.; Clohisy J. C. Aseptic Loosening of Total Joint Replacements: Mechanisms Underlying Osteolysis and Potential Therapies. Arthritis Res. Ther. 2007, 9 (Suppl 1), S6 10.1186/ar2170. PMC1924521 17634145

[ref10] Jung B. A.; Yildizhan F.; Wehrbein H. Bone-to-Implant Contact of Orthodontic Implants in Humans--a Histomorphometric Investigation. Eur. J. Orthod. 2008, 30 (6), 552–557. 10.1093/ejo/cjn054. 19054812

[ref11] Wu H.; Ueno T.; Nozaki K.; Xu H.; Nakano Y.; Chen P.; Wakabayashi N. Lithium-Modified TiO _2_ Surface by Anodization for Enhanced Protein Adsorption and Cell Adhesion. ACS Appl. Mater. Interfaces 2023, 55232 10.1021/acsami.3c06749. 38014813

[ref12] Tian L.; Sheng Y.; Huang L.; Chow D. H.-K.; Chau W. H.; Tang N.; Ngai T.; Wu C.; Lu J.; Qin L. An Innovative Mg/Ti Hybrid Fixation System Developed for Fracture Fixation and Healing Enhancement at Load-Bearing Skeletal Site. Biomaterials 2018, 180, 173–183. 10.1016/j.biomaterials.2018.07.018. 30041069

[ref13] Kawashita M.; Endo N.; Watanabe T.; Miyazaki T.; Furuya M.; Yokota K.; Abiko Y.; Kanetaka H.; Takahashi N. Colloids and Surfaces B: Biointerfaces Formation of Bioactive N-Doped TiO 2 on Ti with Visible Light-Induced Antibacterial Activity Using NaOH, Hot Water, and Subsequent Ammonia Atmospheric Heat Treatment. Colloids Surf. B Biointerfaces 2016, 145, 285–290. 10.1016/j.colsurfb.2016.05.017. 27208442

[ref14] Kokubo T.; Kim H. M.; Kawashita M.; Nakamura T. Bioactive Metals: Preparation and Properties. J. Mater. Sci. Mater. Med. 2004, 15 (2), 99–107. 10.1023/B:JMSM.0000011809.36275.0c. 15330042

[ref15] Kim H.-M.; Miyaji F.; Kokubo T.; Nakamura T. Preparation of Bioactive Ti and Its Alloys via Simple Chemical Surface Treatment. J. Biomed. Mater. Res. 1996, 32 (3), 409–417. 10.1002/(SICI)1097-4636(199611)32:3<409::AID-JBM14>3.0.CO;2-B. 8897146

[ref16] Yamaguchi S.; Takadama H.; Matsushita T.; Nakamura T.; Kokubo T. Cross-Sectional Analysis of the Surface Ceramic Layer Developed on Ti Metal by NaOH-Heat Treatment and Soaking in SBF. J. Ceram. Soc. Jpn. 2009, 117 (1370), 1126–1130. 10.2109/jcersj2.117.1126.

[ref17] Uchida M.; Kim H.; Kokubo T.; Fujibayashi S.; Nakamura T. Structural Dependence of Apatite Formation on Titania Gels in a Simulated Body Fluid. J. Biomed. Mater. Res. - Part A 2003, 64 (1), 164–170. 10.1002/jbm.a.10414. 12483709

[ref18] Yamamoto D.; Kawai I.; Kuroda K.; Ichino R.; Okido M.; Seki A. Osteoconductivity and Hydrophilicity of TiO _**2**_ Coatings on Ti Substrates Prepared by Different Oxidizing Processes. Bioinorg. Chem. Appl. 2012, 2012, 1–7. 10.1155/2012/495218. PMC3535825 23316128

[ref19] Spriano S.; Yamaguchi S.; Baino F.; Ferraris S. A Critical Review of Multifunctional Titanium Surfaces: New Frontiers for Improving Osseointegration and Host Response, Avoiding Bacteria Contamination.. Acta Biomater. 2018, 79, 1–22. 10.1016/j.actbio.2018.08.013. 30121373

[ref20] Kawashita M.; Yokohama Y.; Cui X.; Miyazaki T.; Kanetaka H. In Vitro Apatite Formation and Visible-Light Photocatalytic Activity of Ti Metal Subjected to Chemical and Thermal Treatments. Ceram. Int. 2014, 40 (8 PART B), 12629–12636. 10.1016/j.ceramint.2014.04.109.

[ref21] Uchida M.; Kim H. M.; Kokubo T.; Fujibayashi S.; Nakamura T. Effect of Water Treatment on the Apatite-Forming Ability of NaOH-Treated Titanium Metal. J. Biomed. Mater. Res. 2002, 63 (5), 522–530. 10.1002/jbm.10304. 12209896

[ref22] Chen P.; Nagai A.; Tsutsumi Y.; Ashida M.; Doi H.; Hanawa T. Differences in the Calcification of Preosteoblast Cultured on Sputter-Deposited Titanium, Zirconium, and Gold: Calcification of Preosteoblast on Metals. J. Biomed. Mater. Res., Part A 2016, 104 (3), 639–651. 10.1002/jbm.a.35598. 26488234

[ref23] Ismail M. F.; Islam M. A.; Khorshidi B.; Tehrani-Bagha A.; Sadrzadeh M. Surface Characterization of Thin-Film Composite Membranes Using Contact Angle Technique: Review of Quantification Strategies and Applications. Adv. Colloid Interface Sci. 2022, 299, 102524 10.1016/j.cis.2021.102524. 34620491

[ref24] Chen P.; Aso T.; Sasaki R.; Tsutsumi Y.; Ashida M.; Doi H.; Hanawa T. Micron/Submicron Hybrid Topography of Titanium Surfaces Influences Adhesion and Differentiation Behaviors of the Mesenchymal Stem Cells. J. Biomed. Nanotechnol. 2017, 13 (3), 324–336. 10.1166/jbn.2017.2335. 29381291

[ref25] Chen P.; Aso T.; Sasaki R.; Ashida M.; Tsutsumi Y.; Doi H.; Hanawa T. Adhesion and Differentiation Behaviors of Mesenchymal Stem Cells on Titanium with Micrometer and Nanometer-scale Grid Patterns Produced by Femtosecond Laser Irradiation. J. Biomed. Mater. Res., Part A 2018, 106 (10), 2735–2743. 10.1002/jbm.a.36503. 30055042

[ref26] Zhao L.; Wang H.; Huo K.; Zhang X.; Wang W.; Zhang Y.; Wu Z.; Chu P. K. The Osteogenic Activity of Strontium Loaded Titania Nanotube Arrays on Titanium Substrates. Biomaterials 2013, 34 (1), 19–29. 10.1016/j.biomaterials.2012.09.041. 23046755

[ref27] Zhao Q.; Ueno T.; Chen P.; Nozaki K.; Tan T.; Hanawa T.; Wakabayashi N. Fabrication of Micro-/Submicro-/Nanostructured Surfaces on Ti–Zr Alloy by Varying H2SO4/H2O2 Treatment Conditions and Investigations of Fundamental Properties of a Typical Surface. Surf. Interfaces 2022, 34, 102390 10.1016/j.surfin.2022.102390.

[ref28] Rupp F.; Gittens R. A.; Scheideler L.; Marmur A.; Boyan B. D.; Schwartz Z.; Geis-Gerstorfer J. A Review on the Wettability of Dental Implant Surfaces I: Theoretical and Experimental Aspects. Acta Biomater. 2014, 10 (7), 2894–2906. 10.1016/j.actbio.2014.02.040. 24590162 PMC4041806

[ref29] Hsu H.-C.; Tsou H.-K.; Hsu S.-K.; Wu S.-C.; Lai C.-H.; Ho W.-F. Effect of Water Aging on the Apatite Formation of a Low-Modulus Ti–7.5Mo Alloy Treated with Aqueous NaOH. J. Mater. Sci. 2011, 46 (5), 1369–1379. 10.1007/s10853-010-4929-y.

[ref30] Zhao G.; Schwartz Z.; Wieland M.; Rupp F.; Geis-Gerstorfer J.; Cochran D. L.; Boyan B. D. High Surface Energy Enhances Cell Response to Titanium Substrate Microstructure. J. Biomed. Mater. Res., Part A 2005, 74A (1), 49–58. 10.1002/jbm.a.30320. 15924300

[ref31] Silva I. R. D.; Barreto A. T. D. S.; Seixas R. S.; Paes P. N. G.; Lunz J. D. N.; Thiré R. M. D. S. M.; Jardim P. M. Novel Strategy for Surface Modification of Titanium Implants towards the Improvement of Osseointegration Property and Antibiotic Local Delivery. Materials 2023, 16 (7), 2755 10.3390/ma16072755. 37049048 PMC10095684

[ref32] Primet M.; Pichat P.; Mathieu M. V. Infrared Study of the Surface of Titanium Dioxides. I. Hydroxyl Groups.. J. Phys. Chem. 1971, 75 (9), 1216–1220. 10.1021/j100679a007.

[ref33] Feng B.; Weng J.; Yang B. C.; Qu S. X.; Zhang X. D. Characterization of Surface Oxide Films on Titanium and Adhesion of Osteoblast. Biomaterials 2003, 24 (25), 4663–4670. 10.1016/S0142-9612(03)00366-1. 12951009

[ref34] Fujibayashi S.; Nakamura T.; Nishiguchi S.; Tamura J.; Uchida M.; Kim H.-M.; Kokubo T. Bioactive Titanium: Effect of Sodium Removal on the Bone-Bonding Ability of Bioactive Titanium Prepared by Alkali and Heat Treatment. J. Biomed. Mater. Res. 2001, 56 (4), 562–570. 10.1002/1097-4636(20010915)56:4<562::AID-JBM1128>3.0.CO;2-M. 11400134

[ref35] Anselme K. Osteoblast Adhesion on Biomaterials. Biomaterials 2000, 21 (7), 667–681. 10.1016/S0142-9612(99)00242-2. 10711964

[ref36] Khalili A.; Ahmad M. A Review of Cell Adhesion Studies for Biomedical and Biological Applications. Int. J. Mol. Sci. 2015, 16 (8), 18149–18184. 10.3390/ijms160818149. 26251901 PMC4581240

[ref37] Yamamura K.; Miura T.; Kou I.; Muramatsu T.; Furusawa M.; Yoshinari M. Influence of Various Superhydrophilic Treatments of Titanium on the Initial Attachment, Proliferation, and Differentiation of Osteoblast-like Cells. Dent. Mater. J. 2015, 34 (1), 120–127. 10.4012/dmj.2014-076. 25748468

[ref38] Costa e Silva Filho F.; Conde Menezes G. Osteoblasts Attachment and Adhesion: How Bone Cells Fit Fibronectin-Coated Surfaces. Mater. Sci. Eng., C 2004, 24 (5), 637–641. 10.1016/j.msec.2004.08.036.

[ref39] Bays J. L.; DeMali K. A. Vinculin in Cell–Cell and Cell–Matrix Adhesions. Cell. Mol. Life Sci. 2017, 74 (16), 2999–3009. 10.1007/s00018-017-2511-3. 28401269 PMC5501900

[ref40] Li J.; Wang G.; Wang D.; Wu Q.; Jiang X.; Liu X. Alkali-Treated Titanium Selectively Regulating Biological Behaviors of Bacteria, Cancer Cells and Mesenchymal Stem Cells. J. Colloid Interface Sci. 2014, 436, 160–170. 10.1016/j.jcis.2014.08.053. 25268820

[ref41] Nordin T.; Graf J.; Frykholm A.; Helldén L. Early Functional Loading of Sand-blasted and Acid-etched (SLA) Straumann Implants Following Immediate Placement in Maxillary Extraction Sockets. Clinical and Radiographic Result. Clin. Oral Implants Res. 2007, 18 (4), 441–451. 10.1111/j.1600-0501.2007.01387.x. 17517056

